# Supplementary material for: Musical instrument classifier for early childhood percussion instruments
Source: PLoS One. 2024 Apr 2;19(4):e0299888. doi: 10.1371/journal.pone.0299888 (PMC10986987; doi:10.1371/journal.pone.0299888)
Supplement: S1 Appendix — (PDF) [file pone.0299888.s001.pdf]

**S1 Appendix.** Do It Yourself (DIY) Instruments

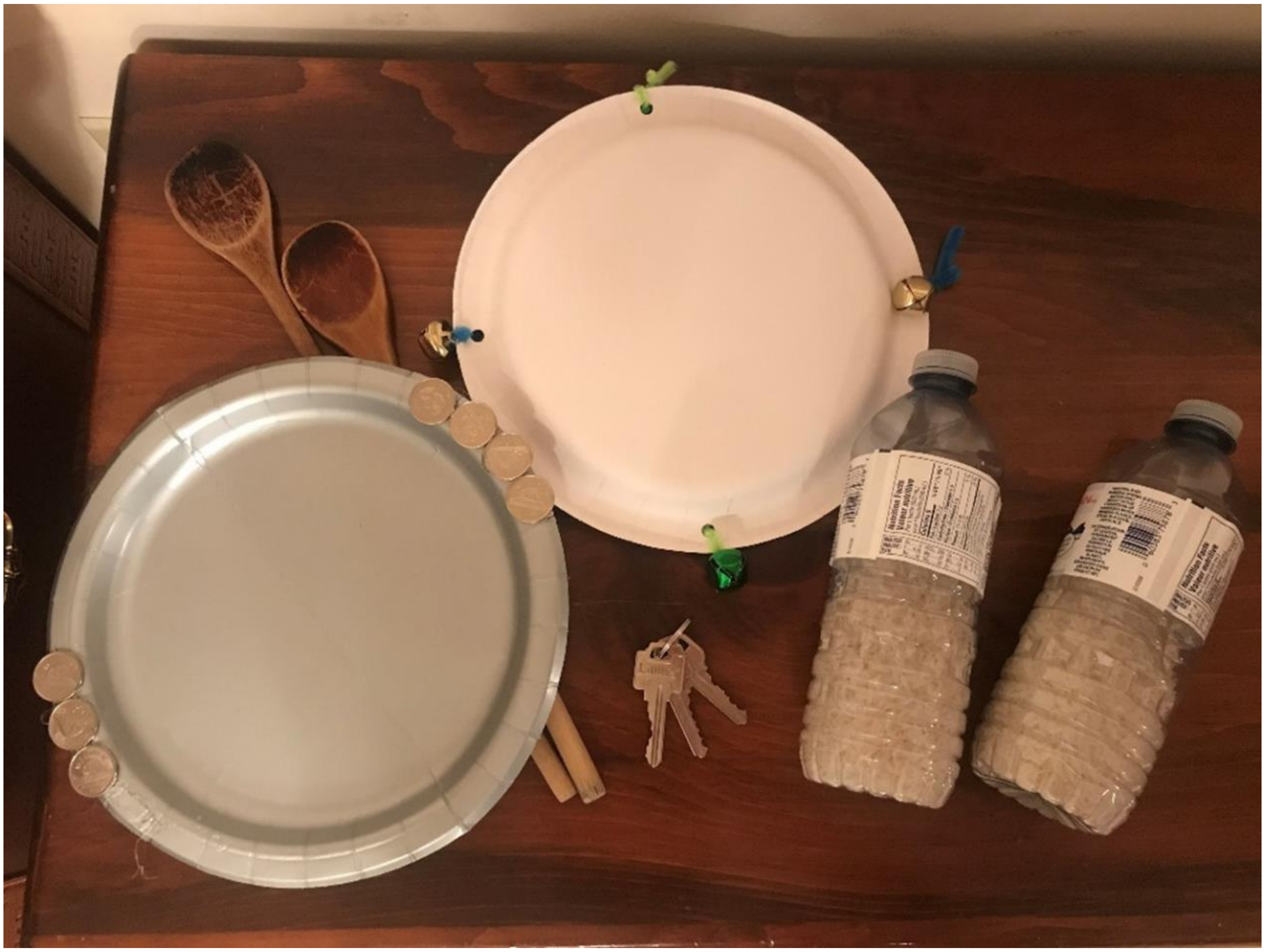

*Reprinted from Pearl Interactives under a CC BY license, with permission from Sharon Wong, CEO of Pearl Interactives, original copyright 2024.*
