# Supplementary material for: Musical instrument classifier for early childhood percussion instruments
Source: PLoS One. 2024 Apr 2;19(4):e0299888. doi: 10.1371/journal.pone.0299888 (PMC10986987; doi:10.1371/journal.pone.0299888)
Supplement: S2 Appendix — Italicized indicates selected features from NCA. (PDF) [file pone.0299888.s002.pdf]

S2 Appendix. List of feature extraction. Italicized indicates selected features from NCA**.**

**Feature**	**Dimension**
Mel-frequency cepstral coefficients	13
Mean absolute deviation	1
Signal mean	1
Signal median	1
Signal standard deviation	1
Inter quantile range	1
Signal quantiles (25th and 75th percentile)	2
Sample skewness	1
Signal entropy	1
Spectral entropy	1
Sample kurtosis	1
Dominant frequency values<br>(max frequency, max magnitude, max ratio)	3
Total	27
