# Supplementary material for: Musical instrument classifier for early childhood percussion instruments
Source: PLoS One. 2024 Apr 2;19(4):e0299888. doi: 10.1371/journal.pone.0299888 (PMC10986987; doi:10.1371/journal.pone.0299888)

S3 Appendix. Spectrogram of castanet (top), tambourine (middle) and shaker (bottom). Parameters: 44.1 kHz sampling rate, 50% overlap Hanning window, and 4096 samples DFT.

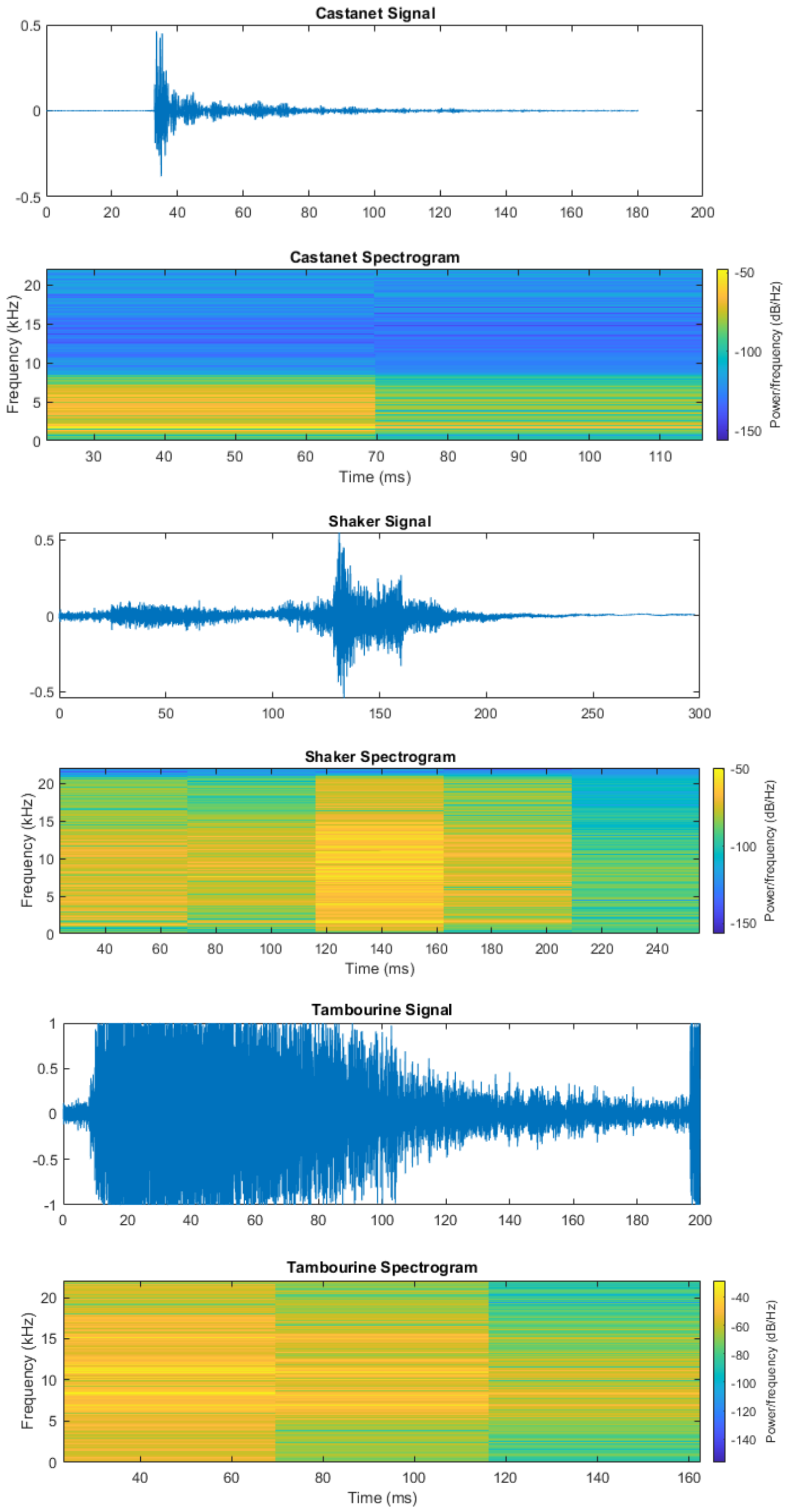

Supplement: S3 Appendix — Parameters: 44.1 kHz sampling rate, 50% overlap Hanning window, and 4096 samples DFT. (PDF) [file pone.0299888.s003.pdf]
