# Supplementary material for: Musical instrument classifier for early childhood percussion instruments
Source: PLoS One. 2024 Apr 2;19(4):e0299888. doi: 10.1371/journal.pone.0299888 (PMC10986987; doi:10.1371/journal.pone.0299888)
Supplement: S4 Appendix — (PDF) [file pone.0299888.s004.pdf]

S4 Appendix. Optimized LGBM parameters using Optuna

Parameter	Value
Number of leaves	135
Number of boosting rounds	1000
Metric	‘multi logloss’
Boosting type	‘gbdt’
Lambda L1	9.57655
Lambda L2	0.473801
Minimum child samples	50
Feature fraction	0.7
Bagging fraction	0.703012
Bagging frequency	6
