# Supplementary material for: Musical instrument classifier for early childhood percussion instruments
Source: PLoS One. 2024 Apr 2;19(4):e0299888. doi: 10.1371/journal.pone.0299888 (PMC10986987; doi:10.1371/journal.pone.0299888)
Supplement: S5 Appendix — Using approximately 93 ms window and reporting macro-average result across all classes. Bold represents best performance. (PDF) [file pone.0299888.s005.pdf]

S5 Appendix. Baseline model results to in-lab test set. Using approximately 93 ms window and reporting macro-average result across all classes. Bold represents best performance.

Model	Precision	Recall	Macro-F1	Accuracy
AdaBoost	0.836	0.827	0.830	0.837
XGBoost	0.814	0.796	0.801	0.810
LGBM	**0.845**	**0.835**	**0.839**	**0.844**
LR	0.669	0.661	0.658	0.676
SVM	0.749	0.754	0.750	0.756
MLP	0.813	0.796	0.802	0.813
KNN	0.800	0.782	0.789	0.796
