# Supplementary figures and images for: Musical instrument classifier for early childhood percussion instruments
Source: PLoS One. 2024 Apr 2;19(4):e0299888. doi: 10.1371/journal.pone.0299888 (PMC10986987; doi:10.1371/journal.pone.0299888)

**S6 Appendix. Confusion matrix analysis for the LGBM model using a 93ms window.**


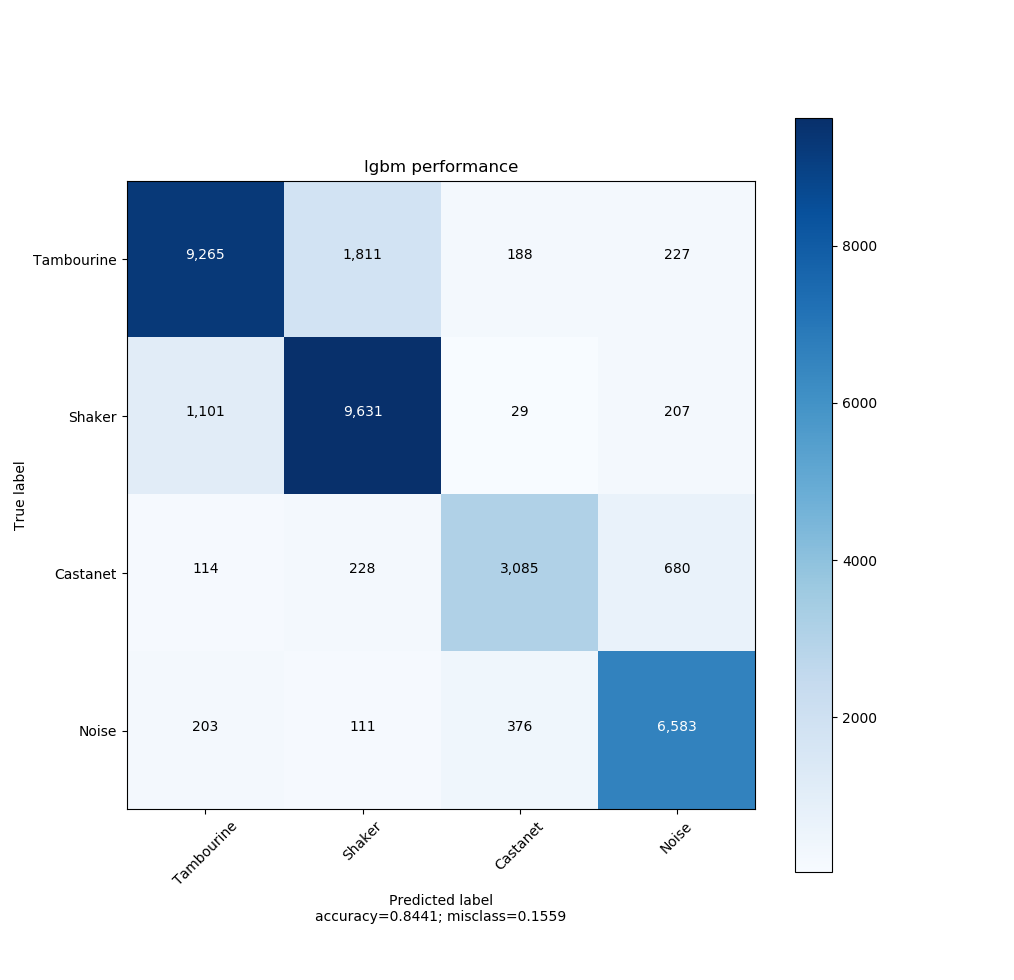

Supplement: S6 Appendix — (DOCX) [file pone.0299888.s006.docx]

**S7 Appendix. SHAP results.** ‘Class 0’ = tambourines, ‘Class 1’ = shakers, ‘Class 2’ = castanets, ‘Class 3’ = noise.

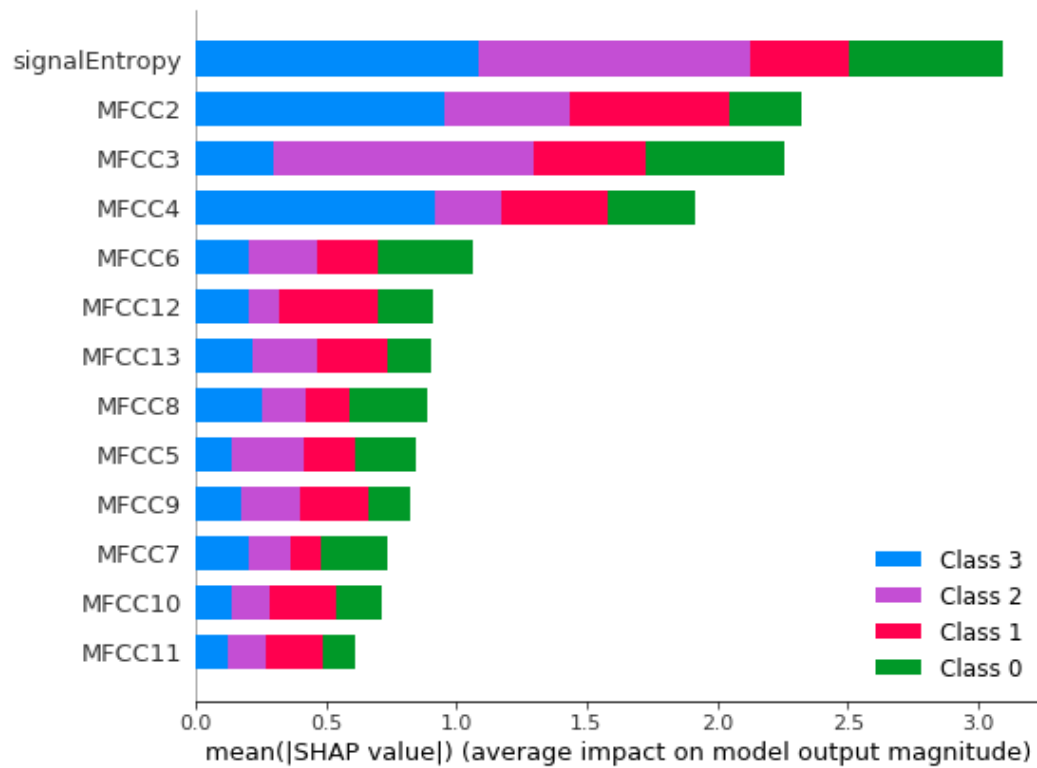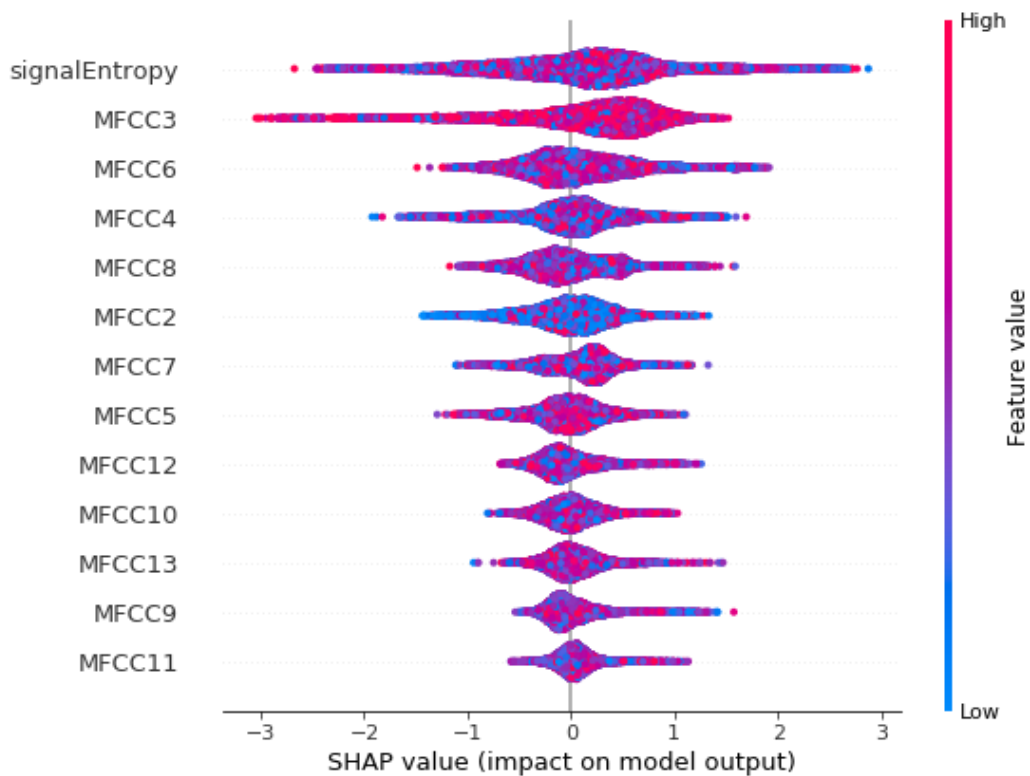

Supplement: S7 Appendix — ‘Class 0’ = tambourines, ‘Class 1’ = shakers, ‘Class 2’ = castanets, ‘Class 3’ = noise. (PDF) [file pone.0299888.s007.pdf]
