# Supplementary material for: Musical instrument classifier for early childhood percussion instruments
Source: PLoS One. 2024 Apr 2;19(4):e0299888. doi: 10.1371/journal.pone.0299888 (PMC10986987; doi:10.1371/journal.pone.0299888)
Supplement: Attachment — Submitted filename: Comments PONE-D-23-33875.docx [file pone.0299888.s009.docx]

1. Please add paper contributions in the abstract.
2. Please add paper organization at the end of the introduction.
3. Please show the data distribution graph.
4. The paper didn’t consider the related work section.
5. Please add the following reference article for your discussion.
   a. Music recommender system via deep learning, Journal of Information and Optimization Sciences 43 (5), 1081-1088.
6. A comparison of other machine learning models is missing.
7. State-of-the-art comparison is not considered in the analysis.
8. Confusion matrix analysis is missing.
9. Computation time analysis is not considered.
